# *AmTH* Plays an Indispensable Role in Larval Development as Well as Adult Immunity in *Apis mellifera*

**DOI:** 10.3390/insects17080821

**Published:** 2026-08-07

**Authors:** Liqiang Liang, Linyan Tian, Shanxin Gao, Shengli Wu, Bo Wei, Yulu Yi, Nanni Ma, Jingwei Luo, Jingwen Sheng, Shangning Yang, Shaohan Tang, Songkun Su, Hongyi Nie

**Affiliations:** 1 College of Animal Sciences/College of Bee Science, Fujian Agriculture and Forestry University, Fuzhou 350002, China; mifengwang122@163.com (L.L.); 52406009025@fafu.edu.cn (L.T.); 12507044002@fafu.edu.cn (S.G.); wushengli2024@163.com (S.W.); 52507057005@fafu.edu.cn (B.W.); yyl@stu.fafu.edu.cn (Y.Y.); 19559160736@163.com (N.M.); 3235602059@stu.fafu.edu.cn (J.L.); 19890587077@163.com (J.S.); shaohan1999@126.com (S.T.); 2 Shandong Provincial Key Laboratory of Animal Cells and Developmental Biology, School of Life Sciences, Shandong University, Qingdao 266237, China; 3 Shandong Provincial Livestock Products Quality and Safety Center, Jinan 250000, China; 4 State Key Laboratory of Resource Insects, Southwest University, Chongqing 400715, China

**Keywords:** *Apis mellifera*, *AmTH*, CRISPR/Cas9, 3-IT, LPS

## Abstract

In insects, tyrosine hydroxylase (TH) plays crucial roles in a wide variety of biological processes. However, its function in *Apis mellifera* remains unknown. Here, *AmTH* (*A. mellifera TH*) was cloned and characterized. Expression profiles at different stages showed that *AmTH* was highly expressed in 2-day-old eggs and during the pupal stage. Tissue expression profiles of newly emerged workers revealed that *AmTH* was highest expressed in the wings, followed by the legs and antennae. To explore the function of *AmTH*, gene-edited worker larvae were generated using CRISPR/Cas9 technology, and the edited individuals died at the larval and prepupal stages. In addition, a TH inhibitor (3-IT) was administered via larval feeding and the survival rates were significantly lower in groups fed with 800 μM, 3200 μM, and 12,800 μM of 3-IT in a dose-dependent manner. To investigate the role of *AmTH* in honeybee immunity, 8-day-old worker bees were fed with 3-IT and subsequently injected with lipopolysaccharide. The results showed that the mRNA expression of *Relish*, antimicrobial peptide-related genes, *TH* and *DDC* were significantly downregulated, whereas *laccase 2* and *yellow-y* were significantly upregulated. These findings indicate that *AmTH* is indispensable for larval development and adult immunity in honeybees.

## Simple Summary

In insects, tyrosine hydroxylase (TH) is the rate-limiting enzyme of the dopamine signaling pathway and governs a series of key biological processes, including development and immunity. In the present study, we investigated the physiological functions of the *TH* gene in the western honeybee (*Apis mellifera*). Spatiotemporal expression profiling demonstrated that *AmTH* was highly expressed in two-day-old eggs and throughout the pupal stage, with its expression peaking in the wing tissues of newly emerged worker bees. Both CRISPR-mediated gene editing and application of TH inhibitors markedly decreased the pupation rate of honeybee larvae. After TH expression was suppressed by inhibitors and the workers were subsequently challenged with lipopolysaccharide, we found that *AmTH* mediates immune responses by modulating the expression of *Relish* and antimicrobial peptides, as well as by activating the melanization pathway. Collectively, these results provide a solid basis for the comprehensive clarification of the physiological roles of *AmTH* in *A. mellifera*.

## 1. Introduction

Dopamine is widely involved in several important biological process from insects to mammals [1]. In insects, the dopamine signaling pathway proceeds as follows: (1) conversion of tyrosine to dopa catalyzed by tyrosine hydroxylase (TH); (2) production of dopamine from dopa via Dopa decarboxylase (DDC); (3) synthesis of N-acetyldopamine or N-β-alanyldopamine from dopamine via N-acylation; (4) conversion of corresponding quinones through oxidation; and (5) pigment production via protein cross-linking [2,3,4]. Dopamine or its derivatives play critical roles in development, immunity, behavior, melanism and other processes in insects [5,6].

TH, serving as the rate-limited enzyme of dopamine pathway, participates in various physiology processes in insects [4]. It plays a conserved role in the epidermal melanization of larvae and pupae. For instance, inhibition of TH via chemical inhibitor or RNA interference caused melanization defects in pupae of *Bactrocera dorsalis*, *Blattella germanica*, *Chilo suppressalis*, *Bombyx mori*, *Plutella xylostella*, *Zeugodacus tau*, *Plagiodera versicolora*, *Spodoptera exigua*, *Platymeris biguttatus* and so on [3,4,7,8,9,10,11,12,13]. In addition, TH plays an indepensable role in the development of egg, larvae and pupae in *Drosophila melanogaster*, and *Nilaparvata lugens* [6,14]. In *Agrotis ipsilon*, individuals injected with Cas9 protein and sgRNA exhibited lower egg-hatching and larval-survival rates compared to the wild type [15]. Furthermore, TH is involved in insect immunity response by regulating the melanin metabolism pathway [11]. Knockdown of *TH* via RNA interference and inhibitor administration led to reduced survival rate upon microbial infection in *Bactrocera dorsalis* and *Plutella xylostella* [4,11].

Although the function of TH has been extensively studied in several insect species, reports on TH gene in *Apis mellifera* remain scarce. Watanabe et al. demonstrated that mRNA expression level of *AmTH* was significantly higher in the brain of 4-day-old male honeybees than in 0-day-old males, and that juvenile hormone analogs could upregulate its expression [16]. Emery et al. found that colonization by the gut symbiont *Frischella perrara* upregulated *TH* expression as revealed by transcriptome method [17]. Additionally, TH protein distribution has been observed in the brain of foragers using immunohistochemistrym [18]. To date, existing studies on AmTH have primarily focused on brain dopamine metabolism in drones and transcriptional responses to gut symbionts, with no systematic investigation linking *AmTH* to larval metamorphosis or systemic antibacterial immunity. Consequently, the comprehensive physiological function of *AmTH* gene in *A. mellifera* remains largely unexplored.

Here we propose a hypothesis to resolve this research gap: *AmTH* is an indispensable upstream factor of honeybee dopamine signaling, which is required for normal embryonic development and larval-pupal metamorphosis; during bacterial infection, *AmTH* positively activates the Imd immune cascade by modulating the expression of *Relish* and downstream antimicrobial peptides, while coordinating the melanization pathway to resist pathogen invasion. Genetic knockout or chemical inhibition of AmTH will trigger developmental lethality at larval/prepupal stages and drastically suppress adult antibacterial immune responses.

To systematically verify this hypothesis, we integrated bioinformatic characterization, spatiotemporal expression profiling, CRISPR/Cas9 gene editing, chemical inhibitor feeding, and bacterial immune challenge assays. Briefly, we first cloned and analyzed the evolutionary conservation of AmTH, and then mapped its developmental and tissue-specific expression patterns. We generated *AmTH* loss-of-function mutants via embryo microinjection of sgRNA-Cas9 complexes and applied the TH inhibitor 3-IT to quantify larval survival and pupation defects. Finally, we challenged adult workers with LPS and *E. coli* to dissect how *AmTH* modulates key immune and melanization genes. Collectively, our results confirm that *AmTH* is indispensable for larval development and adult immunity in *A. mellifera*.

## 2. Materials and Methods

### 2.1. Colonies and Sample Collection

Samples of Fengqiang No. 1 *A. mellifera* were collected from the apiary of the College of Bee Science at Fujian Agriculture and Forestry University (26.08° N, 119.30° E). Colonies were established using queens that had undergone natural mating within the past year. To accurately collect experimental materials at different developmental stages, egg laying was restricted to a 3 h period by confining queen, following established methods [19]. The collected samples encompassed the following developmental stages: eggs at 1–3 days old (E1–E3), larvae at 1, 3, and 5 days old (L1, L3, L5), prepupae at 1 and 3 days old (PP1, PP3), and pupae at 2, 4, 6, and 8 days old (P2, P4, P6, P8), as well as newly emerged bees (NEBs), nurse bees, and forager bees. All samples were immediately placed on dry ice upon collection. For developmental stage expression profiling, each biological replicate consisted of 100 individuals for E1, E2, E3, and L1; 50 individuals for L3; and 5 individuals for all remaining stages. For tissue-specific expression analysis, NEBs were dissected to separate the head, thorax, abdomen, gut, stinger, antennae, legs, and wings, with each biological replicate comprising 10 individuals for head, thorax, and abdomen, and 20 individuals for the remaining tissues (antennae, legs, wings, gut, and stinger). Three biological replicates, each derived from a genetically distinct colony, were collected for each sampling point (or tissue) for subsequent RNA extraction, and all qPCR reactions were performed in technical triplicates.

### 2.2. Cloning and Bioinformatics Analysis

The CDS of *AmTH* was amplified by PCR using cDNA from NEBs as the template. The gene-specific primers used for amplification were *AmTH*-F (5′-ATGATGGCTGTAGCAGCGG-3′) and *AmTH-R* (5′-TTACGCGAACGATGTCTTCAG-3′). The PCR reaction was performed in a total volume of 50 μL, containing: 25 μL of 2 × Phanta Max Buffer, 1 μL of Phanta Max Super-Fidelity DNA Polymerase (Vazyme Biotech, Nanjing, China), 1 μL of dNTP Mix (10 mM each), 2 μL each of forward and reverse primers (10 μM), 5 μL of cDNA template, and 14 μL of ddH_2_O. The PCR program was as follows: initial denaturation at 95 °C for 3 min; 35 cycles of denaturation at 95 °C for 15 s, annealing at 60 °C for 15 s, and extension at 72 °C for 1 min; followed by a final extension at 72 °C for 5 min. The PCR product was purified, ligated into a Blunt Zero vector (TransGen Biotech, Beijing, China), transformed into Trans1-T1 competent cells, and then sent to a biotechnology company for sequencing.

The amino acid sequence encoded by *AmTH* was predicted using BioEdit software (v7.2.6). The physicochemical properties of the protein were analyzed with the online tool ProtParam (https://web.expasy.org/protparam/), accessed on 10 October 2025; its hydrophobicity was predicted using ProtScale (https://web.expasy.org/protscale/), accessed on 10 October 2025 and the signal peptide was predicted with SignalP 6.0 (https://services.healthtech.dtu.dk/services/SignalP-6.0/), accessed on 10 October 2025. Meanwhile, sequences homologous to the *A. mellifera* TH protein were downloaded from the NCBI database for insects belonging to Hymenoptera, Diptera, Coleoptera, Hemiptera, Lepidoptera, and Orthoptera (the accession numbers of these homologous sequences are listed in Table S1). A phylogenetic tree of TH proteins from 29 insect species was constructed using the maximum likelihood (ML) method in MEGA 11.0 software. The best-fit amino acid substitution model was determined using the built-in model-selection function, with the Bayesian Information Criterion (BIC) as the selection metric. The Jones–Taylor–Thornton (JTT) model [20] was identified as the optimal model for our protein sequence dataset and was subsequently employed for ML tree construction. Nodal support was evaluated using 1000 bootstrap replicates. All remaining parameters were maintained at their default settings.

### 2.3. Quantitative Real-Time PCR (qPCR)

The total RNA was extracted from each sample using the Trizol (TransGen Biotech, Beijing, China), and 1 μg of RNA was reverse-transcribed into cDNA. The resulting cDNA served as the template for qPCR, with *A. mellifera actin* used as the internal reference gene. Primers for immune-related and melanin-pathway genes were derived from previously published reports [21,22,23,24], and all primer sequences used are listed in Table S2. Each qPCR reaction was performed in a 10 μL system containing 5 μL of 2× ChamQ SYBR Color qPCR Master Mix (Vazyme Biotech, Nanjing, China), 0.2 μL each of forward and reverse primers, 1 μL of cDNA, and 3.6 μL of ddH_2_O. The thermal cycling conditions were as follows: initial denaturation at 95 °C for 3 min, followed by 39 cycles of 95 °C for 10 s and 60 °C for 30 s. Melting curve analysis was conducted from 65 °C to 95 °C with increments of 0.5 °C every 5 s. Relative expression levels of target genes were calculated using the 2^−ΔΔCt^ method [25]. All quantitative analyses were performed with three biological replicates and three technical replicates. Statistical analysis was carried out using GraphPad Prism version 5, and significance was evaluated by one-way ANOVA or independent-samples *t*-test.

### 2.4. Microinjection of sgRNA Targeting the AmTH Gene in A. mellifera

According to the 5‘-GGGN20NGG-3′ targeting rule, a 23 bp target site was selected within *AmTH*. Following the method described by Bassett et al. [26], the sgRNA transcription template was synthesized by annealing two complementary primers (F: TTAATACGACTCACTATAGGGGCTTGTGCTGCGCCTGAGAGGTTTTAGAGCTAGAAATAG; R: AGCACCGACTCGGTGCCACTTTTTCAAG). The underlined fragment is the sgRNA targeting sequence of *AmTH*. Subsequently, based on the established protocol of our group [21], eggs laid within 1.5 h were collected and arranged in an orderly manner. A mixture of sgRNA targeting *AmTH* gene (500 ng/μL) and Cas9 protein (500 ng/μL) was microinjected into the eggs, while the control group was injected with an equal volume of ddH_2_O. After injection, the eggs were promptly transferred to an incubator (temperature: 34.5 °C, humidity: 85%) until larval hatching. The rearing of worker bee larvae and the preparation of their diet were carried out according to the method described by Kaftanoglu et al. [27]. For comparisons of proportions (hatching rate and pupation rate) between the experimental and control groups, Fisher’s exact test was applied. A significance threshold of *p* < 0.05 was used for all statistical analyses.

### 2.5. Genomic DNA Extraction and Detection of Mutagenesis

Genomic DNA was extracted from prepupae or pupae using the phenol–chloroform method, following the protocol described by Bi et al. [28]. A fragment encompassing the CRISPR-targeted site within *AmTH* was amplified by PCR using the primers *AmTH*-mut-F (5′-ATTCATCCAGACGCTGGACTGAC-3′) and *AmTH*-mut-R (5′-TGATGTGACGAATGTACTGAGTGCTC-3). The resulting PCR products were ligated into the Blunt-Zero vector (TransGen Biotech, Beijing, China), followed by transformation and plating. For each individual, ten randomly selected colonies were subjected to Sanger sequencing of the target site to identify mutations induced by CRISPR/Cas9 editing [29].

### 2.6. Feeding of the 3-Iodotyrosine (3-IT) in A. mellifera

As one inhibitor of TH, 3-iodotyrosine (3-IT) was widely used to explore the biology function in various insect species [4,11,30]. To investigate the function of AmTH during development, we fed larvae of *A. mellifera* with 3-IT. For 2-day-old larvae, they were continuously fed diets supplemented with different concentrations (200, 800, 3200, 12,800 μM) of the TH inhibitor 3-IT during the larval stage, while the control group received an equal volume of ddH_2_O. The larvae were maintained in an incubator (35 °C, 90% humidity). The diet composition and feeding amounts for larvae of different ages followed established protocols from the literature [27]. The number of surviving bees was recorded daily, and survival curves were plotted. The number of daily deaths was recorded. Survival curves were generated using GraphPad Prism, and statistical significance was assessed using the Log-rank (Mantel–Cox) test with Bonferroni-corrected pairwise comparisons.

### 2.7. Lipopolysaccharide (LPS) Injection into A. mellifera Adult Workers

NEBs were collected and housed in plastic rearing cages (30 bees per cage). Three independent biological replicates were performed, with each replicate consisting of bees derived from a distinct colony and housed in a separate cage. They were maintained in an incubator (30 °C, 40% relative humidity) and provided with a 50% sucrose solution and fresh pollen. The sucrose solution and pollen were replenished daily. When the bees reached 8 days old, they were anesthetized with CO_2_. Subsequently, each bee was injected with 5 µL of LPS (2.2 µg/µL) into the intersegmental membrane between the third and fourth abdominal segments. The control group received an equal volume of phosphate-buffered saline (PBS). After injection, the bees were returned to the incubator. Samples were collected one hour later to measure the relative expression level of *AmTH* in *A. mellifera*.

To further validate the relationship between *TH* expression and LPS-induced immune challenge, a verification experiment involving dietary administration of the inhibitor 3-IT was conducted. Similarly, NEBs were collected and housed in plastic rearing cages (30 bees per cage, three independent biological replicates) under the same temperature and humidity conditions. For the first 7 days, all bees were fed a 50% sucrose solution and fresh pollen daily. Starting on day 8, the experimental group was switched to a 50% sucrose solution supplemented with the 3-IT at a final concentration of 10,000 µM, while the control group continued to receive the 50% sucrose solution without the inhibitor. The sucrose solution was replaced daily. After this dietary treatment for 3 days, bees from both groups were anesthetized with CO_2_. Bees in the experimental group were then injected with 5 µL of LPS, and the control group was injected with an equal volume of PBS. Samples were collected one hour after injection to determine the relative expression levels of *Relish*, antimicrobial peptide genes, and key genes in the melanization pathway.

### 2.8. E. coli Injection in Adult A. mellifera

Sealed brood combs nearing emergence were selected from the bee colony and placed in a constant-temperature incubator (30 °C, 76% relative humidity). NEBs were collected every 24 h. Following the method described by Scharlaken et al. [31], a 5 μL suspension of *E. coli* was injected into the intersegmental membrane between the third and fourth abdominal segments of newly emerged bees using a microinjection pump, while the control group received an equal volume of PBS buffer. Each group consisted of 60 bees. After injection, the bees were transferred to a constant-temperature incubator for continued rearing. Subsequently, the relative expression levels of the *AmTH* were detected at three time points—2 h, 4 h, and 8 h post-infection with *E. coli*—using quantitative real-time PCR.

## 3. Results

### 3.1. Cloning and Bioinformatic Analysis of AmTH in A. mellifera

**Figure 1:**
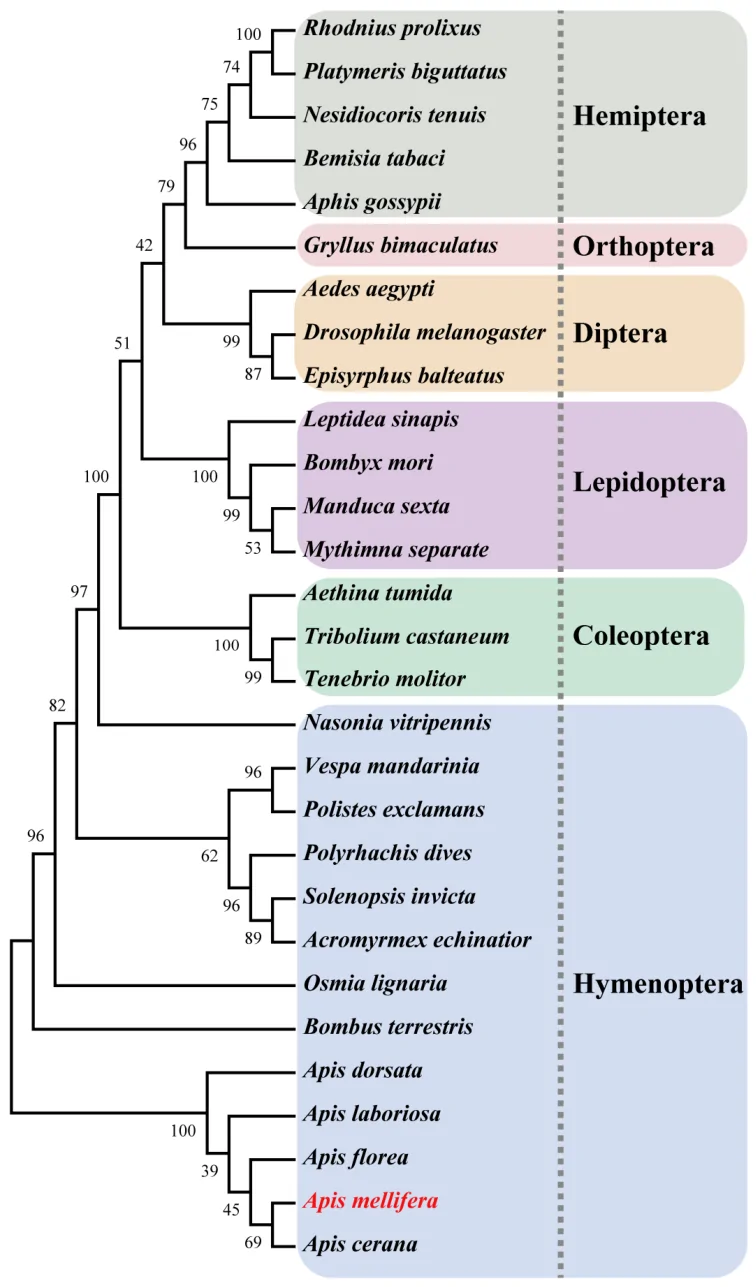
Phylogenetic tree of tyrosine hydroxylase (TH) proteins in various insects.

Using cDNA from newly emerged bees of *A. mellifera* as a template, a 1683 bp CDS sequence of *AmTH* was amplified by PCR. After TA cloning and sequencing, the obtained sequence was consistent with the NCBI reference sequence (Gene ID: 408930). ProtParam analysis predicted that the AmTH protein consists of 560 amino acids, with an isoelectric point (pI) of 5.55 and a molecular weight of approximately 63.8 kDa. The protein lacks a signal peptide and is likely intracellular. Hydrophobicity analysis indicated that the amino acid at position 257 of the AmTH protein had the lowest score (−3.089), while the amino acid at position 247 had the highest score (1.700), suggesting that the protein is hydrophilic overall. Furthermore, domain analysis revealed that the AmTH protein contains a Tyr_3_monoox domain, located between amino acids 48 and 506. Phylogenetic analysis revealed that the TH amino acid sequences from Hymenoptera, Coleoptera, Lepidoptera, Diptera, Orthoptera, and Hemiptera each clustered into distinct monophyletic branches (Figure 1). Within this tree, TH from *A. mellifera* showed the closest evolutionary relationship to that of the *Apis cerana* in Hymenoptera. The amino acid sequence of AmTH from *A. mellifera* shared 71.32–99.8% identity with other insect TH homologs (Table S3). Among hymenopterans, the similarity ranges from 83.99% (in *Nasonia vitripennis*) to 99.8% (in *Apis cerana*).

### 3.2. Spatiotemporal Expression Patterns of AmTH in A. mellifera

**Figure 2:**
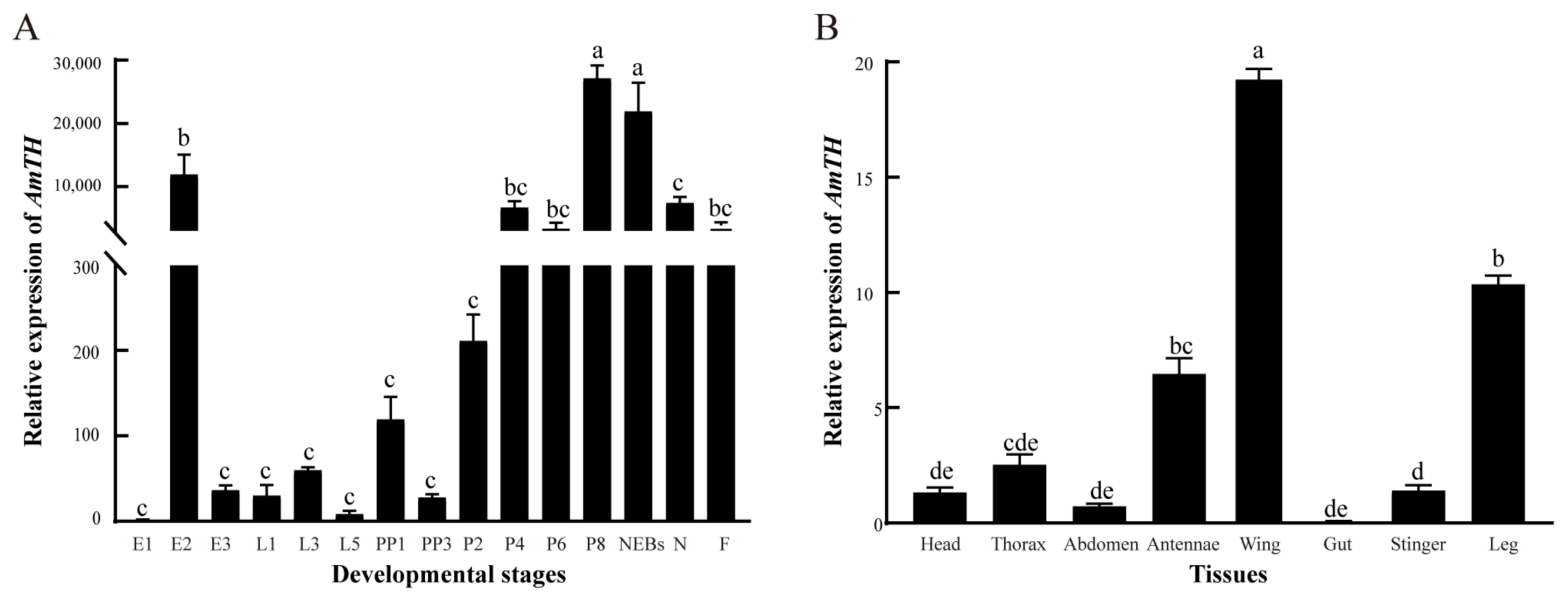
Expression profile of *AmTH* across developmental stages and in various tissues of newly emerged bees (NEBs). (**A**) Temporal expression pattern of the *AmTH* gene. E1, E2 and E3 denote 1-, 2-, and 3-day-old eggs, respectively. L1, L3 and L5 indicate 1-, 3-, and 5-day-old larvae, respectively. PP1 and PP3: 1-, 3-day-old pharate pupae; P2, P4, P6, P8: 2-, 4-, 6-, and 8-day-old pupae, respectively; NEBs: newly emerged bees; N: nurses; F: foragers. (**B**) Tissue-specific expression of *AmTH* in NEBs. Samples include the head (without antennae), thorax, abdomen (excluding stinger, venom gland and gut), gut, stinger (with venom gland), antennae, wing, and leg. Statistical analyses were performed using one-way ANOVA followed by Tukey’s HSD test and distinct lowercase letters denote significant differences (*p* < 0.05).

Developmental expression profiling revealed that *AmTH* exhibited dynamic changes (Figure 2A). It was extremely low in 1-day-old eggs, increased rapidly and peaked in 2-day-old eggs, then decreased markedly in 3-day-old eggs. Throughout the larval stage, its expression remained relatively low. Expression increased again during the prepupal stage. Upon pupation, *AmTH* expression continuously rose starting from 2-day-old pupae, peaking significantly at 8-day-old pupae, and gradually declined in subsequent stages. Expression in adult bees decreased relative to peak pupal levels yet remained moderately high. Tissue expression profiling in NEBs showed pronounced tissue-specific expression of *AmTH* (Figure 2B). The transcript level was significantly highest in wings, followed by legs and antennae. Moderately lower expression was detected in the stinger, thorax, and head, while expression was nearly undetectable in the gut.

### 3.3. CRISPR/Cas9-Mediated Mutation of AmTH in A. mellifera

**Figure 3:**
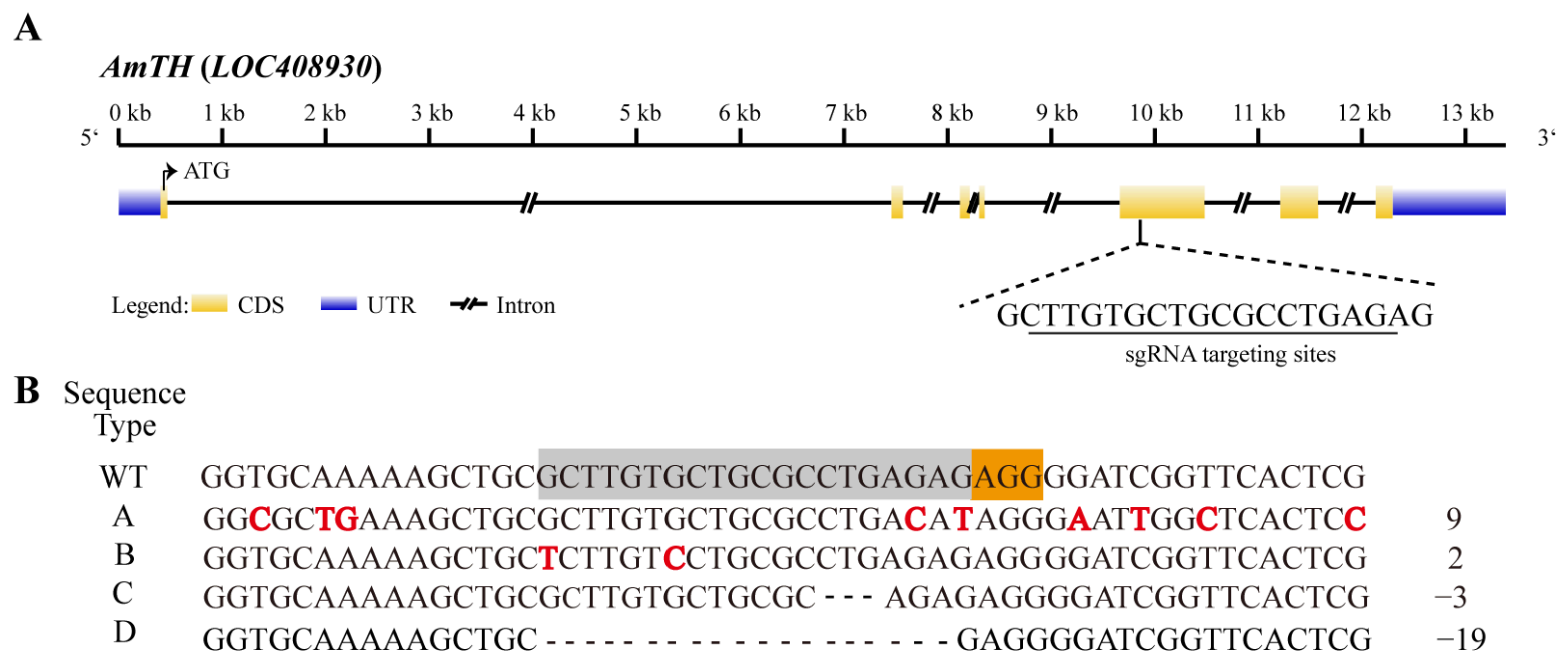
CRISPR/Cas9-mediated mutagenesis analysis of the *AmTH* gene in *A. mellifera*. (**A**) Design of the sgRNA target site in the *AmTH*. (**B**) Types of mutations induced by CRISPR/Cas9 at the target site. Note: The target site is highlighted in gray; the PAM sequence is indicated in orange. Dashed lines represent deleted nucleotides, and red font denotes nucleotide substitutions. Numbers to the right of each sequence indicate length changes (“−”, deletion).

We designed a specific sgRNA target site within exon 5 of *AmTH* in *A. mellifera* (Figure 3A) and coinjected a mixture of the sgRNA and Cas9 protein into 352 eggs within 1.5 h after oviposition. The hatching rate in the experimental group was 11.93% (42/352), which was significantly lower than that in the control group (65.82%, 52/79; Fisher’s exact test, *p* < 0.0001). Among the hatched individuals, eight in the experimental group reached the prepupal stage, with four (50.00%) successfully pupating, whereas 34 in the control group reached the prepupal stage, with 32 (94.12%) successfully pupating; this difference was also statistically significant (Fisher’s exact test, *p* = 0.0084). TA cloning and sequencing analysis revealed that no mutations were detected in the four successfully pupated individuals from the experimental group. In contrast, the four individuals that failed to pupate showed various deletions and substitutions near the target site (Figure 3B), which we categorized into four types (A–D) based on their mutational outcomes (Figure S1 and Table S4): Type A had nine nucleotide substitutions causing premature termination at position 96; Type B had two synonymous substitutions with no amino acid change; Type C had a 3 bp deletion resulting in leucine loss at position 94; and Type D had a 19 bp deletion leading to premature termination at position 99. These results suggest that *AmTH* participates in the larval hatching and pupation process.

### 3.4. Feeding with the 3-IT Affects Larvae Development and Pupation in A. mellifera

**Figure 4:**
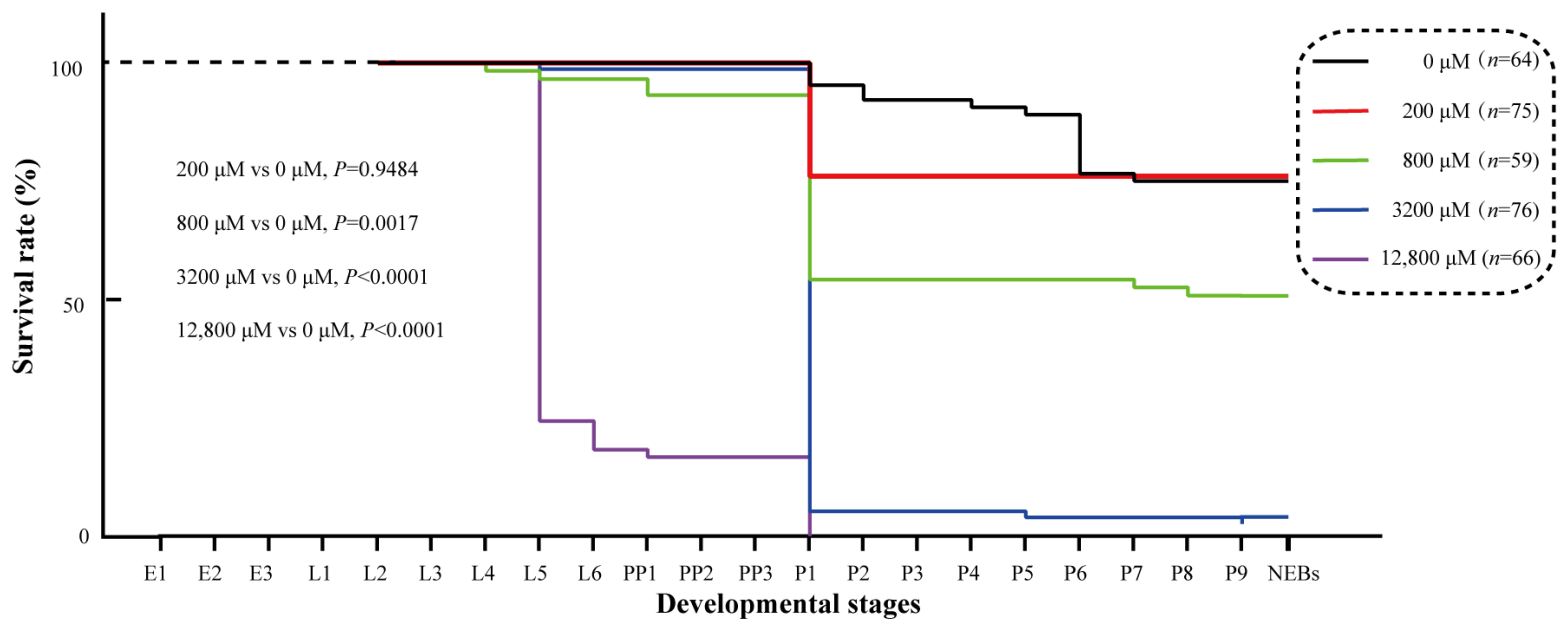
Survival curves of *A. mellifera* workers that were fed larval food containing 3-IT. The concentrations of 3-IT were set as 0 μM (control, *n* = 64), 200 μM (n = 75), 800 μM (*n* = 59), 3200 μM (*n* = 76), and 12,800 μM (*n* = 66), where *n* represents the number of larvae initially used in each treatment group. Developmental stages are denoted as follows: E1–E3, 1- to 3-day-old eggs; L1–L6, 1- to 6-day-old larvae; PP1, PP2 and PP3, 1-, 2- and 3-pharate pupae; P1–P9, 1- to 9-day-old pupae; NEBs, newly emerged bees. Survival curves were compared using the Log-rank (Mantel–Cox) test with Bonferroni-corrected pairwise comparisons. After Bonferroni correction, the adjusted pairwise *p*-value was 0.0125. Differences between the two groups were considered statistically significant when the *p*-value was less than 0.0125.

To further investigate the role of the *AmTH* in the pupation process of *A. mellifera*, we treated larvae starting from 2 days old with varying concentrations of the 3-IT to examine the impact of *AmTH* inhibition on survival rate (Figure 4). According to the results, the overall survival curves showed a statistically significant difference (χ^2^ = 332.9, *p* < 0.0001). The results showed that larvae fed with 200 μM 3-IT exhibited no significant difference in survival rate compared to the control group (χ^2^ = 0.0042, *p* = 0.9484). In contrast, survival rates were significantly lower in groups fed with 800 μM (χ^2^ = 9.873, *p* = 0.0017), 3200 μM (χ^2^ = 110.9, *p* < 0.0001), and 12,800 μM (χ^2^ = 142.1, *p* < 0.0001) of 3-IT compared to the control group, and a dose-dependent effect was observed between the feeding concentration and survival rate. Furthermore, for the 200, 800, and 3200 μM treatment groups, a significant proportion of larval mortality occurred specifically during the prepupal stage. Notably, at the highest concentration of 12,800 μM, none of the larvae developed to the pupal stage. Consistent with phenotype from CRISPR/Cas9 knockout, these results further demonstrate that *AmTH* is critical for pupation in *A. mellifera*.

### 3.5. TH Induces Immune Responses in Adult Honeybees

**Figure 5:**
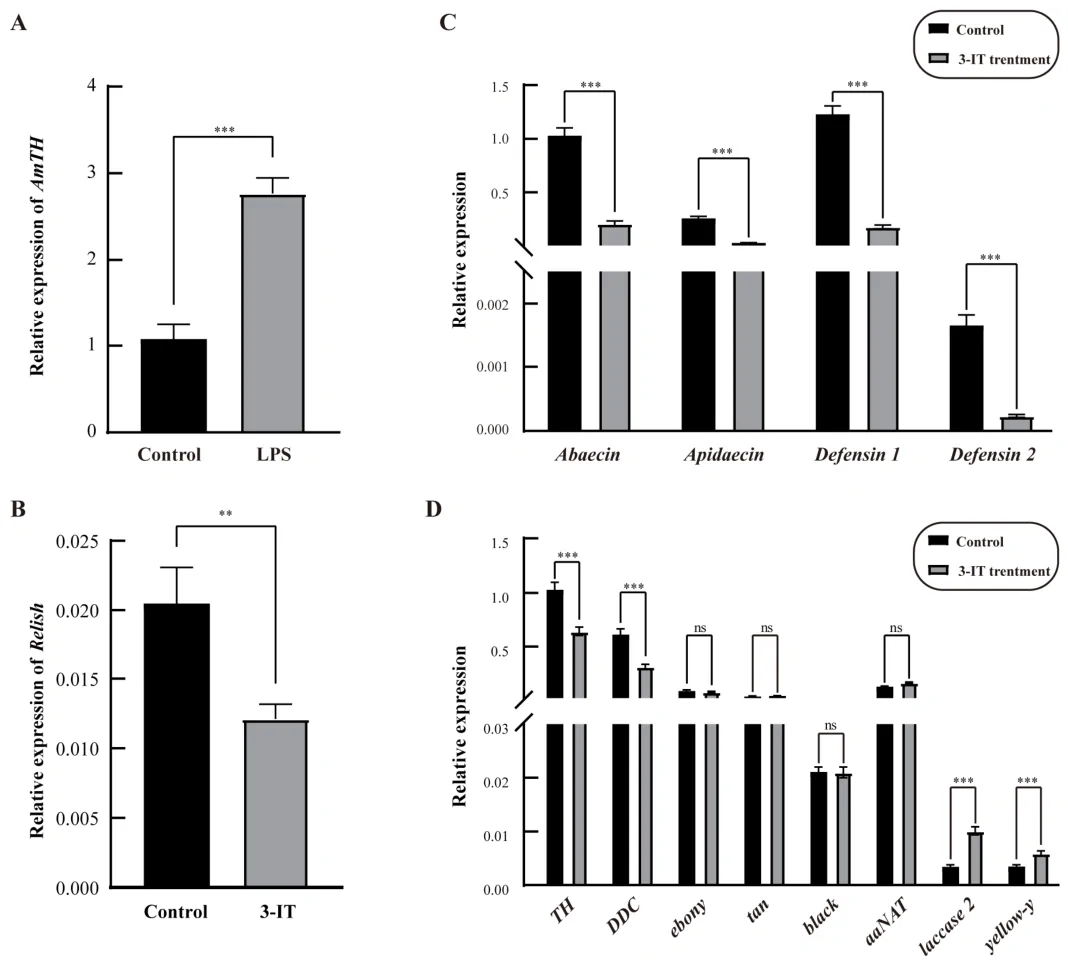
The influence of *AmTH* on the immune response in adult worker honeybees (*A. mellifera*). (**A**) Relative expression level of *AmTH* in 8-day-old adult worker bees in response to LPS injection. Changes in the expression of immune-related genes in adult worker bees following tyrosine hydroxylase Inhibitor (3-IT) feeding and LPS injection: *Relish* (**B**), antimicrobial peptide genes (**C**), and key melanization pathway genes (**D**). Data are presented as mean ± standard error. Significance analysis of the relative gene expression was performed using independent-samples *t*-test; ** indicates *p* < 0.01, *** indicates *p* < 0.001, and “ns” indicates no significant difference.

Lipopolysaccharide (LPS), as a pathogen-associated molecular pattern, could be used to trigger immune responses in *A. mellifera* [23]. To investigate its role in the honeybee immune system, we injected LPS into 8-day-old adult bees and measured the expression level of the *AmTH* via qPCR 1 h later. The results demonstrated significant upregulation of *AmTH* in the LPS-injected group, with expression levels approximately 2.6-fold higher than those in the control group (Figure 5A). Consistently, *AmTH* expression was also markedly elevated in NEBs at 2, 4, and 8 h after *E. coli* injection compared to the control (Figure S2), indicating that the upregulation of *AmTH* persists over a broader time window upon bacterial challenge. Collectively, these findings suggest that *AmTH* participates in honeybee immune response.

To further explore the underlying mechanism, and to ensure the consistency and reproducibility of the immune challenge across all replicates, we continuously fed 8-day-old bees with 3-IT-supplemented sugar water for three days prior to LPS injection. Subsequently, we examined the expression profiles of multiple genes involved in immune responses and melanin synthesis. qPCR analysis revealed that *Relish* expression in the experimental group significantly decreased to about 60% of control levels (Figure 5B). The expression of antimicrobial peptide genes (*Abaecin*, *Apidaecin*, *Defensin-1*, *Defensin-2*) was significantly downregulated (Figure 5C). Among core melanin synthesis genes, expression of *TH* and *DDC* was significantly reduced, whereas *laccase 2* and *yellow-y* was significantly upregulated (Figure 5D).

## 4. Discussion

In *Agrotis ipsilon*, *TH*-editing individuals exhibited an embryo-hatching rate of only 14.5%, compared with 84.6% in the control group [15]. In *Bombyx mori*, RNAi-mediated knockdown of *TH* similarly blocked embryo hatching [32]. In our study, the expression profiles at different stages showed that *AmTH* was highly expressed in 2-day-old eggs (Figure 2A), suggesting that *AmTH* mediates mid-embryonic development in *A. mellifera*. Furthermore, in the experimental group injected with sgRNA and Cas9 protein, the embryo-hatching rate was only 11.93%, whereas the ddH_2_O-injected control reached 65.82%. These results demonstrate that the role of TH in insect embryonic development is relatively conserved and that *AmTH* also plays a key role in honeybee embryogenesis. In addition, relatively high *AmTH* mRNA expression levels were observed during the prepupal stage in *A. mellifera* (Figure 2A), suggesting that *AmTH* was involved in metamorphosis process. In our CRISPR experiments, eight individuals developed to the prepupal stage. Among these, four successfully pupated, and we detected no mutations in the 10 TA clones sequenced per individual. By contrast, the four individuals that failed to reach the pupal stage showed various mutation patterns (Types A–D) (Figure 3B and Table S4). We acknowledge, however, that the 10-clone sampling strategy has limited sensitivity for detecting low-frequency mosaic alleles in diploid honeybee workers. Therefore, the absence of detectable mutations in the pupated individuals does not definitively rule out the presence of rare edited alleles. Meanwhile, the dose-dependent reduction in pupation rate observed in the 3-IT feeding assays independently corroborates the phenotypic defects observed in CRISPR-edited individuals (Figure 4). Collectively, these two orthogonal approaches provide compelling evidence that *AmTH* plays an essential role in the pupation process of *A. mellifera*. A significant decrease in pupation rate following inhibition of TH has also been observed in other insects, indicating that the function of the TH in regulating insect pupation is conserved across different insect species [4,7]. The 20-Hydroxyecdysone (20E) is a critical modulator of pupating in insects [33]. Bai et al. found that *GbTH* knockout reduced the 20E titer by repressing the expression of *NPC1b*, a gene involved in lipid transport, thereby leading to developmental deficiency in *Gryllus bimaculatus* [34]. Therefore, we speculate that *AmTH* regulate metamorphosis in *A. mellifera* via a conserved mechanism similar to that in *Gryllus bimaculatus*. Interestingly, tissue expression profiling of NEBs showed that *AmTH* is most highly expressed in the wings (Figure 2B). Similarly, in *Euschistus heros*, individuals with dsRNA injection exhibited deformed wing [35], and in *Bombyx mori*, treatment with a TH inhibitor repressed adult wing development [13]. These findings suggest that *AmTH* may also play a role in wing development in *A. mellifera*; however, the underlying mechanism remains unknown.

When we injected adult workers with either LPS or *E. coli*, the mRNA level of *AmTH* was significantly upregulated (Figure 5A and Figure S2). This upregulation demonstrates that both LPS and *E. coli* challenges trigger immune responses dependent on *AmTH*. This finding is consistent with previous reports on TH in *Manduca sexta* [36]. Furthermore, 3-IT treatment resulted in a marked downregulation of the mRNA levels of transcription factor *Relish* and antimicrobial peptide genes (AMPs) including *Abaecin*, *Apidaecin*, *Defensin 1* and *Defensin 2* (Figure 5B,C). In *A. mellifera*, RNA interference-mediated knockdown of *Relish* downregulated the expression of *Abaecin* and *Hymenoptaecin*, but did not affect *defensin-1* [23]. Moreover, *Relish* can directly activate certain AMPs by binding to their promoter in several insects and aquatic organism [37,38,39]. Based on these observations, we propose that 3-IT suppress *Abaecin* and *Apidaecin* primarily through reducing *Relish* expression. By contrast, the mechanism by which 3-IT affected *Defensin 1* and *Defensin 2* remains unclear and warrants further investigation. The suppression of AMPs expression upon TH inhibition is widely conserved across insect taxa [4,11]. To further elucidate how *AmTH* participated in honeybee immunity beyond AMP regulation, we examined several core genes involved in melanin biosynthesis following 3-IT treatment. In the 3-IT treatment group, *TH* and *DDC* mRNA levels were significantly reduced, while *laccase2* and *yellow-y* were strongly upregulated. We hypothesize that elevated *laccase2* and *yellow-y* represent a compensatory feedback response within the tyrosine metabolism cascade. Consistent with this possibility, 3-IT treatment also upregulates downstream genes in the TH pathway in *Plutella xylostella* and *Bactrocera dorsalis* [4,11]. Collectively, these results demonstrate that *AmTH* repression of *TH* affects not only *Relish* and AMP expression, but also multiple critical genes in the melaninization pathway. We therefore conclude that *AmTH* is indispensable for immunity response in *A. mellifera*, a finding that aligns with previous reports on the role of TH in immunity across various insect species [4,11,40].

Based on the phylogenetic tree, TH protein sequences are highly conserved among different insects. Previous studies have extensively documented that TH serve as a critical regulator of embryo hatching and larvae development in pests, such as *Agrotis ipsilon*, *Drosophila melanogaster*, *Gryllus bimaculatus* and *Bombyx mori* [6,15,32,41]. In our study, we observed significantly higher egg and larvae mortality in *AmTH* mutant individuals than in wild-type individuals. Similarly, survival rates were markedly lower in groups fed with 800 μM, 3200 μM, and 12,800 μM of 3-IT than the control group (Figure 4). These results are consistent with previous findings in other insects, indicating that not only protein sequence but also physiology functions are highly conserved across different insect species. Given this functional conservation, we propose that TH represents a promising target for gene drive-based pest control, as has been widely reported in several mosquito species [42,43,44,45]. Notably, similar effects on egg hatching and larval survival have also been observed in honeybees following TH knockout or inhibition. Because honeybees are economically vital pollinators, we recommend that any TH-targeted biocontrol strategy should select target sequences that are divergent between pest species and honeybee (e. g., *Apis mellifera* or *Apis cerana*) to minimize off-target harm to pollinator populations.

## 5. Conclusions

In this study, we comprehensively characterized the spatiotemporal expression patterns of *AmTH* in *Apis mellifera*. Using CRISPR/Cas9 gene knockout and TH inhibitor feeding, we confirmed that *AmTH* is essential for larval development and pupation. Furthermore, we demonstrated that *AmTH* drives immune responses by upregulating the antimicrobial peptide expression and activating the melanization pathway. These results provide an important basis for comprehensively elucidating the physiological functions of *AmTH* in *A. mellifera*.

## Data Availability

All data generated or analyzed during this study are included in this published article.

## Supplementary Materials

The following supporting information can be downloaded at https://www.mdpi.com/article/10.3390/insects17080821/s1, Figure S1: Four mutational types (A–D) in *AmTH* genome-edited individuals exhibit distinct outcomes at the protein level; Figure S2: *AmTH* expression in *A. mellifera* in response to *E. coli* infection; Table S1: Accession numbers for tyrosine hydroxylase genes from different insects; Table S2: Primers used in this study; Table S3: Percent identity matrix for the comparison of amino acid sequence identities of tyrosine hydroxylase homologs from various insects; Table S4: Summary of Sanger sequencing results from 10 TA clones per individual.
